# NANOMATERIALS: Transformation of Silver Nanoparticles in Sewage Sludge

**DOI:** 10.1289/ehp.118-a526a

**Published:** 2010-12

**Authors:** Carol Potera

**Affiliations:** **Carol Potera**, based in Montana, has written for *EHP* since 1996. She also writes for *Microbe*, *Genetic Engineering News*, and the *American Journal of Nursing*

The release and environmental fate of nanoparticles throughout the life cycle of “nanoenabled” goods is an area of growing research interest. In the first known field study of the fate of silver nanoparticles in the wastewater treatment system, researchers now report these nanoparticles transform into silver sulfide in the sludge produced by sewage treatment plants.^1^ This new information about the life cycle of silver nanoparticles provides a starting point for further exploring their impact on the environment.

Silver has been used as an antimicrobial agent for millennia,^2^ and the increased surface area offered by the nanoparticle form of the metal offers greater germ-killing capacity.^3^ Today, manufacturers add silver nanoparticles to hundreds of consumer products, including food storage containers, clothing, computer keyboards, cosmetics, pillows, cell phones, and medical appliances.^4^

Silver is water soluble, so contact with any type of moisture—such as a bath or a spin in the washing machine—washes some out and sends it into wastewater systems. “We wanted to know what form of silver enters the environment after it goes down the drain and passes through sewage treatment plants,” says Michael Hochella, a geochemist at Virginia Polytechnic Institute and State University and director of natural and incidental nanoparticles for the multi-institute Center for the Environmental Implications of NanoTechnology.^5^

Sludge from sewage treatment facilities can end up as landfill or soil amendments in agricultural fertilizers, or it can be burned in incinerators. In 2006 and 2007 the U.S. Environmental Protection Agency (EPA) analyzed sewage sludge samples from 74 municipal wastewater treatment facilities nationwide and tested for 28 metals, including silver (which was detected in all the samples).^6^ Through the EPA, Hochella and postdoctoral fellow Bojeong Kim obtained frozen samples of sludge from a Midwest facility. They suspected it would contain the nanosilver particles now used in consumer products—although the EPA’s goal in sampling was simply to obtain national estimates of the concentrations of selected analytes, not identify nanoparticles.

Kim developed analytical methods to determine the size, chemistry, and atomic structure of silver nanoparticles in the samples. The samples tested high in silver, but the silver could not be attributed to an industrial source. Scanning transmission electron microscopy revealed the nanoparticles were 5–20 nm in diameter and formed small, loosely packed aggregates no more than 100 nm in size. Energy-dispersive X-ray spectrometry showed that sulfur (which is produced by microorganisms that digest sewage) combined with the silver in a 2:1 ratio, and the crystal structure confirmed the formation of silver sulfide nanoparticles.^1^

The results underscore the complexity of environmental fate. “What we start with is not what ends up in the environment,” Hochella says. The researchers don’t know how many silver nanoparticles were introduced to the wastewater treatment plants or how much incoming nanosilver ended up as silver sulfide nanoparticles. However, Kim notes that no pure silver nanoparticles were found in the sludge.

In general, silver sulfide is highly insoluble and settles out of water.^7^ But no one knows if silver sulfide nanoparticles behave in the same way. Properties of metals can change dramatically as particle size decreases.^3^ “It’s hard to predict whether the solubility of nanoparticles will increase, decrease, or stay the same,” Kim says. The bioavailability, toxicity, and reactivity of silver sulfide nanoparticles also are unknown.

If silver sulfide nanoparticles do prove toxic, the environmental implications could be unfavorable. Antimicrobial nanoparticles could adversely impact desirable microorganisms that decompose waste in sewage treatment plants, says Murray McBride, director of the Cornell Waste Management Institute. Furthermore, McBride says, nanosized silver sulfide applied to agricultural land could oxidize in soils and release toxic silver ions that kill beneficial soil microorganisms. On the other hand, one study of laboratory-grown *Pseudomonas putida* biofilms indicated some bacteria bind silver ions, potentially rendering them less toxic.^8^

## Figures and Tables

**Figure:**
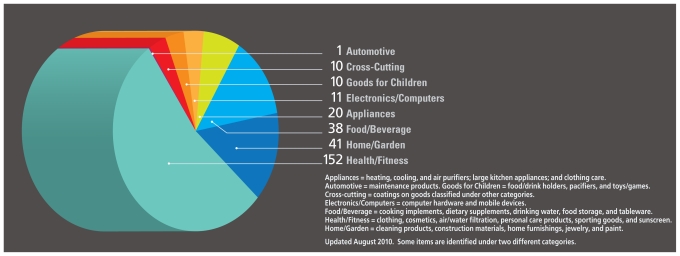
Numbers of Goods Containing Silver Nanoparticles^4^
